# Chronic Lead Exposure Decreases the Vascular Reactivity of Rat Aortas: The Role of Hydrogen Peroxide

**DOI:** 10.1371/journal.pone.0120965

**Published:** 2015-03-25

**Authors:** Karolini Zuqui Nunes, Dieli Oliveira Nunes, Edna Aparecida Silveira, Camila Almenara Cruz Pereira, Gilson Brás Broseghini Filho, Dalton Valentim Vassallo, Mirian Fioresi

**Affiliations:** 1 Department of Physiological Sciences, Federal University of Espírito Santo, Vitória, ES, Brazil; 2 Department of Nursing, Federal University of Espírito Santo, Vitória, ES, Brazil; 3 Health Science Centre of Vitória—EMESCAM, Vitória, Espírito Santo, Brazil; University of Arkansas for Medical Sciences; College of Pharmacy, UNITED STATES

## Abstract

We investigated whether exposure to small concentrations of lead alters blood pressure and vascular reactivity. Male Wistar rats were sorted randomly into the following two groups: control (Ct) and treatment with 100 ppm of lead (Pb), which was added to drinking water, for 30 days. Systolic blood pressure (BP) was measured weekly. Following treatment, aortic ring vascular reactivity was assessed. Tissue samples were properly stored for further biochemical investigation. The lead concentration in the blood reached approximately 8 μg/dL. Treatment increased blood pressure and decreased the contractile responses of the aortic rings to phenylephrine (1 nM–100 mM). Following N-nitro-L arginine methyl ester (L-NAME) administration, contractile responses increased in both groups but did not differ significantly between them. Lead effects on R_max_ were decreased compared to control subjects following superoxide dismutase (SOD) administration. Catalase, diethyldithiocarbamic acid (DETCA), and apocynin increased the vasoconstrictor response induced by phenylephrine in the aortas of lead-treated rats but did not increase the vasoconstrictor response in the aortas of untreated rats. Tetraethylammonium (TEA) potentiated the vasoconstrictor response induced by phenylephrine in aortic segments in both groups, but these effects were greater in lead-treated rats. The co-incubation of TEA and catalase abolished the vasodilatory effect noted in the lead group. The present study is the first to demonstrate that blood lead concentrations well below the values established by international legislation increased blood pressure and decreased phenylephrine-induced vascular reactivity. The latter effect was associated with oxidative stress, specifically oxidative stress induced via increases in hydrogen peroxide levels and the subsequent effects of hydrogen peroxide on potassium channels.

## Introduction

Lead is a common industrial and environmental pollutant that exerts a variety of acute and chronic toxic effects [1]. The metal is used extensively in the industrial sector, which contributes to its wide environmental distribution [2]. In recent years, manufacturers of batteries have emerged as the largest consumer of lead, using approximately 80% of the lead produced globally. The manufacturing and recycling of these products may result in sufficient lead exposure to cause the development of acute and chronic health problems [3].

Chronic lead exposure may trigger a cascade of events that culminates in the development of hypertension and cardiovascular disease [1]. Navas-Acien and collaborators (2007) [4] reviewed the link between lead exposure and cardiovascular events in several population studies, highlighting the elevation of arterial pressure. However, the cardiovascular effects of lead are not limited to increases in blood pressure. Several reports have indicated that lead treatment can either increase or decrease aortic ring vascular reactivity to phenylephrine in response to different conditions and stimuli [5,6,7,8].

Several mechanisms have been proposed as explanations for the vascular changes induced by lead, including changes in NO bioavailability and Na^+^/K^+^-ATPase functional activity [8], increases in the levels of reactive oxygen species and COX-derived contractile prostanoids, and increases in renin-angiotensin activity [5]. However, lead’s effects on human health depend on blood levels and exposure duration.

Using the same experimental model, we demonstrated that chronic exposure to lead promotes cardiac changes in animals with blood lead levels below the limits recommended by international legislation. Furthermore promotes increased systolic blood pressure, diastolic blood pressure and heart rate [9]. However, the vascular effects of exposure to low concentrations of lead over a short time period are not fully understood. Therefore, the present study aimed to evaluate vascular impairment resulting from exposure to low concentrations of lead over a period of 30 days, and to investigate the mechanisms that may be involved in vascular alterations caused by this metal.

## Materials and Methods

### Animals and treatment model

Male Wistar rats were used for this study in accordance with the Guide for the Care and Use of Laboratory Animals, and the study was approved by the Ethics Committee of the Federal University of Espirito Santo (002/2013 CEUA-UFES). During treatment, rats had free access to tap water and were fed standard chow *ad libitum*. When the rats reached two months of age, they were divided into the following two groups: the control group received distilled water for 30 days, and the lead-treated group received 100 ppm of lead acetate in drinking water for 30 days. Following treatment, the rats were anesthetized with urethane (1.2 g/kg, ip). The rats’ thoracic aortas were carefully dissected out, and connective tissue was removed. For the vascular reactivity experiments, the aortas were divided into cylindrical segments: each segment was 4 mm in length. Other segments of the aorta were stored at -80°C until they were needed for a protein expression analysis.

### Blood pressure measurements

Indirect systolic blood pressure was measured at the beginning and end of treatment using tail-cuff plethysmography (IITC Life Science, Inc, Woodland Hills, CA, USA). Conscious rats were restrained for 5–10 min in a warm and quiet room and conditioned to numerous cuff inflation-deflation cycles by a trained operator. Systolic blood pressure was measured, and the mean of three measurements was recorded [10].

### Blood lead level measurements

The lead concentrations of both groups were measured by atomic absorption spectrometry at the Hermes Pardini Laboratory using whole blood samples following 30 days of treatment.

### Vascular reactivity measurements

Aortic segments (4 mm in length) were mounted in an organ bath at 37°C containing 5 mL Krebs-Henseleit solution (in mM: NaCl 118, KCl 4.7, NaHCO_3_ 25, CaCl_2_–2H_2_O 2.5, KH_2_PO_4_ 1.2, MgSO_4_–7H_2_O 1.2, glucose 11 and ethylenediamine-tetra acetic acid—EDTA 0.01) that was continuously gassed with a 95% O_2_ and 5% CO_2_ mixture (pH 7.4). Arterial segments were stretched to an optimal resting tension of 1.0 g. Isometric tension was recorded using a force displacement transducer (TSD125C, CA, USA) connected to an acquisition system (MP100A, BIOPAC System, Inc., Santa Barbara, USA).

Following a 45 min equilibration period, all aortic rings were exposed to two doses of 75 mM KCl (30 min): the first dose was given to check their functional integrity, and the second dose was given to assess maximal induced tension. Endothelial integrity was subsequently tested with acetylcholine (10 μM) using segments previously contracted by phenylephrine (1 μM). Relaxation equal to or greater than 90% represented a positive demonstration of the functional integrity of the endothelium. After a washout period (30 min), increasing concentrations of phenylephrine (10^-10^ M—3.10^-4^ M) were applied. A concentration—response curve was generated, and tension was measured once a plateau was reached. The influence of the endothelium on the response to phenylephrine in both untreated and lead-treated rats was investigated following its mechanical removal, which was accomplished by rubbing the vessel lumen with a needle. The absence of the endothelium was confirmed by the inability of 10 mM acetylcholine (ACh) to induce relaxation.

The effects of the following drugs were evaluated: the nonspecific NOS inhibitor, N-nitro-L arginine methyl ester (L-NAME, 100 μM); the hydrogen peroxide scavenger, catalase (1000 U.mL-1); superoxide dismutase (SOD) (150 U/mL); an SOD blocker, diethyldithiocarbamic acid (DETCA, 0.5 mM); an NADPH oxidase inhibitor, apocynin (0.3 μM,); and the nonselective blocker of K^+^ channels, tetraethylammonium (TEA, 2 mM).

Another set of experiments involved the pre-contraction of aortic rings from both untreated and lead-treated rats with phenylephrine (1 μM) and the subsequent generation of concentration-response curves to either acetylcholine (0.1 nM—300 μM) or sodium nitroprusside (0.01 nM—0.3 μM).

### Western blot analyses

The arteries were rapidly frozen and kept at -80°C until needed for protein expression analyses of both catalase and superoxide dismutase in both the control and lead-treated groups. Proteins from homogenized arteries were separated using 12.5 or 10% SDS-PAGE gels. Proteins were transferred to nitrocellulose membranes and were subsequently incubated overnight at 4°C with anti-catalase (1:100; Sigma Aldrich, St. Louis, MO, USA) and anti-SOD (1:500; Sigma Aldrich, St. Louis, MO, USA) antibodies. Following washing, the membranes were incubated with secondary anti-mouse or anti-rabbit antibodies (1:5000; Stress Gen, Victoria, Canada) conjugated to horseradish peroxidase. Following a thorough washing, the immune complexes were detected using an enhanced horseradish peroxidase/luminal chemiluminescence system (ECL Plus, Amersham International, Little Chalfont, UK) and film (Hyperfilm ECL International). The immunoblot signals were quantified using the ImageJ computer program. The same membrane was used to determine anti-α-actin expression using mouse monoclonal antibodies (1:5000; Sigma, USA).

### Drugs and reagents

Lead acetate Pb (CH_3_COO)_2_, L-phenylephrine hydrochloride, acetylcholine chloride, sodium nitroprusside, N-nitro-L arginine methyl ester, catalase, superoxide dismutase, diethyldithiocarbamic acid, apocynin and tetraethylammonium were purchased from Sigma-Aldrich (Sigma Chemical Co., USA). The salts and reagents used were of analytical grade and were from Sigma-Aldrich and Merck (Darmstadt, Germany).

### Statistical analyses

Contractile responses were expressed as percentages of the maximal response induced by 75 mM KCl. For each concentration-response curve, the maximal effect (R_max_) and the agonist concentration that produced 50% of the maximal response (log EC50) were calculated using nonlinear regression analysis (GraphPad Prism, GraphPad Software, Inc., San Diego, CA, USA). Agonist sensitivities are expressed as pD2 (-log EC50). In order to compare the effects of drugs on the aortic ring responses to phenylephrine, some results were expressed as differences in the areas under the concentration-response curves (dAUC) in both the control and experimental groups. AUCs were calculated from individual concentration-response curve plots: the differences are expressed as the percentage of the control AUC. For protein expression, the data are expressed as the ratio between signals on the immunoblot corresponding to the protein of interest and α-actin. Two-way ANOVA was performed for the blood pressure data analysis, followed by a Bonferroni test. The results are expressed as means ± SEM for the number of rats indicated: differences were analyzed using the unpaired Student t test. P < 0.05 was considered statistically significant.

## Results

No differences in body weight were observed between the groups before (untreated: 259 ± 1.30 g, n = 30; lead-treated: 258 ± 0.99 g, n = 30; P<0.05) or after treatment (untreated: 309 ± 2.56 g, n = 30; lead-treated: 310 ± 2.59 g, n = 30; P<0.05).

In rats exposed to thirty-day lead treatment, the blood lead concentration was 8.4 μg/dL ± 1.1 μg/dL (n = 5). A significant rise in systolic arterial blood pressure was observed seven days following lead exposure (first day: 105 ± 6.8 mmHg, seventh day: 128 ± 4.9 mmHg, n = 8; P<0.05) and was maintained until the 28th day of treatment (126± 2.7 mmHg, n = 8; P<0.05). In the control animals, the pressure remained unchanged during treatment (107 ± 5.0 mmHg, n = 6; P<0.05).

### Effects of lead treatment on vascular reactivity

**Fig 1:**
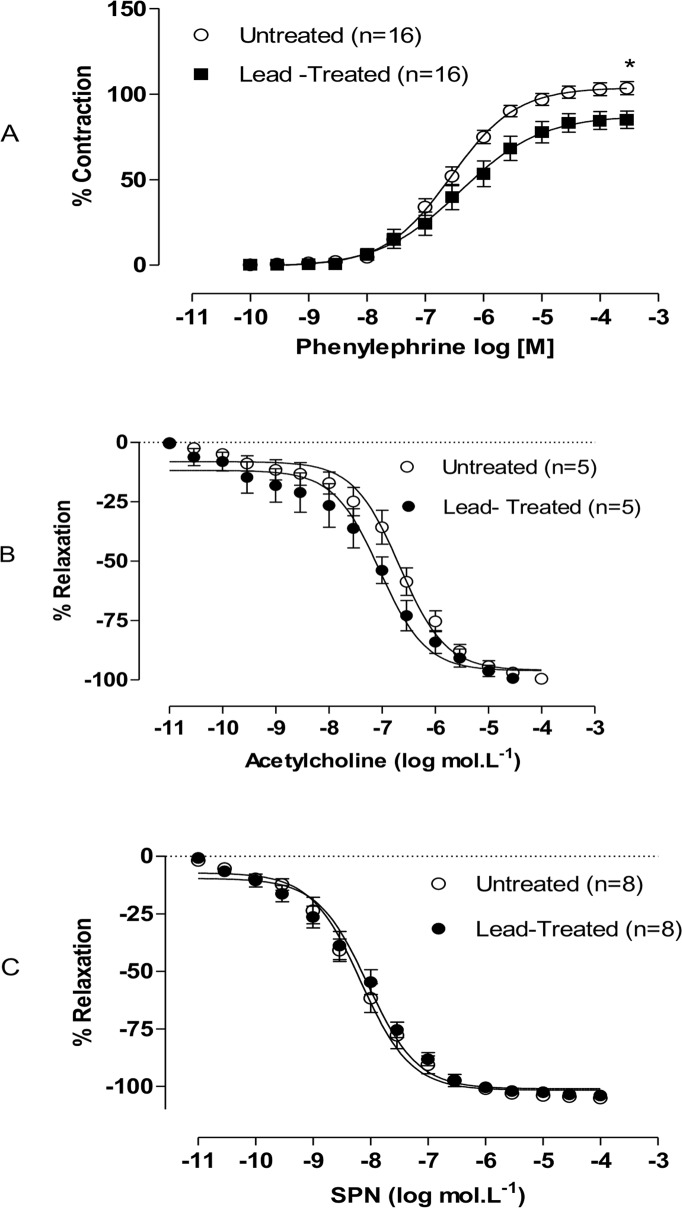
Effects of exposure to lead on the concentration-response curves of phenylephrine, acetylcholine, and sodium nitroprusside. Effects of 30 days of exposure to lead acetate on the concentration-response curves of (A) phenylephrine, (B) acetylcholine, and (C) sodium nitroprusside (SNP) treatment of aortic rings. Each point represents the mean ± SEM. *p < 0.05 by Student’s *t* test. *p < 0.05 versus the corresponding control by Student’s t-test. The number of animals used is indicated in parentheses.

Lead treatment did not affect the response to KCl (untreated: 2.192, n = 30; lead treated: 2.221, n = 30; P>0.05), but it decreased the contractile responses induced by phenylephrine in rat aortas (Fig. 1A). It also decreased R_max_, but not the sensitivity to phenylephrine (Fig. 1A). The concentration-dependent relaxation induced by ACh in the treated group was not different than the response observed in the untreated group (R_max_ untreated: -100 ± 1.8, n = 7; R_max_ lead-treated: -101 ± 1.8, n = 7; pD2 untreated: -6.67 ± 0.06, n = 7; pD2 lead-treated: -7.04 ± 0.09, n = 7). Similarly, the response induced by SNP was the same in both groups (R_max_ untreated: -101 ± 1.3, n = 8; R_max_ lead-treated: -100 ± 0.7, n = 8; pD2 untreated: -8.16 ± 0.05, n = 8; pD2 lead-treated: -8.05 ± 0.05, n = 8) (Fig. 1B, 1C).

### Participation of endothelial factors in the vasodilator response to phenylephrine

**Fig 2:**
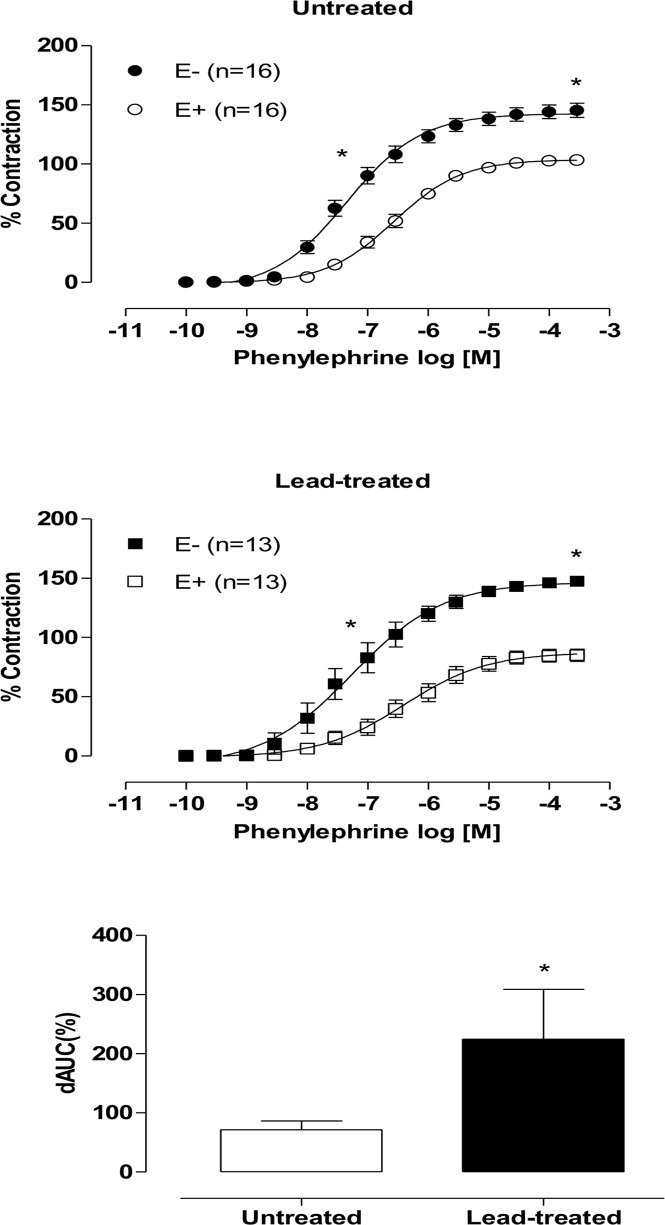
Effects of endothelium removal (E-) in aortic rings from both untreated and lead-treated rats. Effects of endothelium removal (E-) in phenylephrine induced vasoconstriction in aortic rings from both untreated and lead-treated rats. The graph demonstrates differences in the areas under the concentration-response curves (dAUC) in both endothelium-denuded and intact segments. Each point represents the mean ± SEM. *p < 0.05 versus the corresponding control by Student’s t-test. The number of animals used is indicated in parentheses.

To evaluate the influence of the endothelium on vascular responses, we mechanically removed the endothelium. Phenylephrine reactivity following endothelial damage was increased in both experimental groups (Fig. 2), but this increase was superior in the lead-treated group, as demonstrated by the dAUC values (Fig. 2). These data associated with decreased vascular reactivity to phenylephrine suggest that treatment for thirty days with lead acetate causes the release of an endothelium-derived relaxation factor.

**Fig 3:**
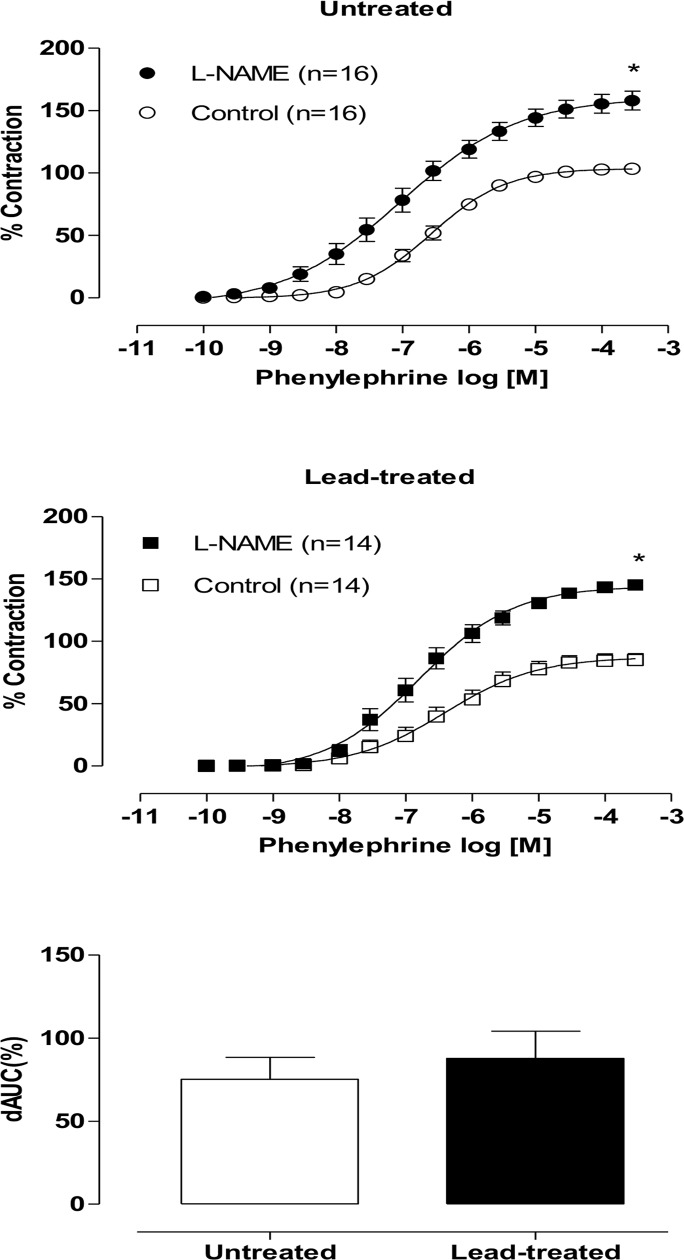
Effects of L-NAME (100 mM) treatment in aortic rings from both untreated and lead-treated rats. Effects of L-NAME (100 mM) treatment on phenylephrine-induced vasoconstriction in aortic rings from both untreated and lead-treated rats. The graph demonstrates that there are no differences in the areas under the concentration-response curves (dAUC) between the groups. Each point represents the mean ± SEM. *p < 0.05 versus the corresponding control by Student’s t-test. The number of animals used is indicated in parentheses.

To evaluate whether lead acetate treatment altered NO-induced modulation of phenylephrine-induced contractile responses, aortic rings were incubated with the NOS inhibitor, L- NAME (100 mM). L-NAME caused similar increases in the maximum response to phenylephrine in arteries from both the control and lead-treated groups (Fig. 3). However, dAUC values demonstrated that the role played by nitric oxide in the contractile response to phenylephrine did not differ between the groups (Fig. 3).

### Participation of reactive oxygen species in the vasodilator response to phenylephrine

Several studies have shown that lead exposure induces the generation of reactive oxygen species (ROS), which subsequently results in oxidative damage to several organ systems and alterations to antioxidant defence systems [11,12,13,14]. Among ROS, hydrogen peroxide acts as an endothelium-derived hyperpolarizing agent [15].

**Fig 4:**
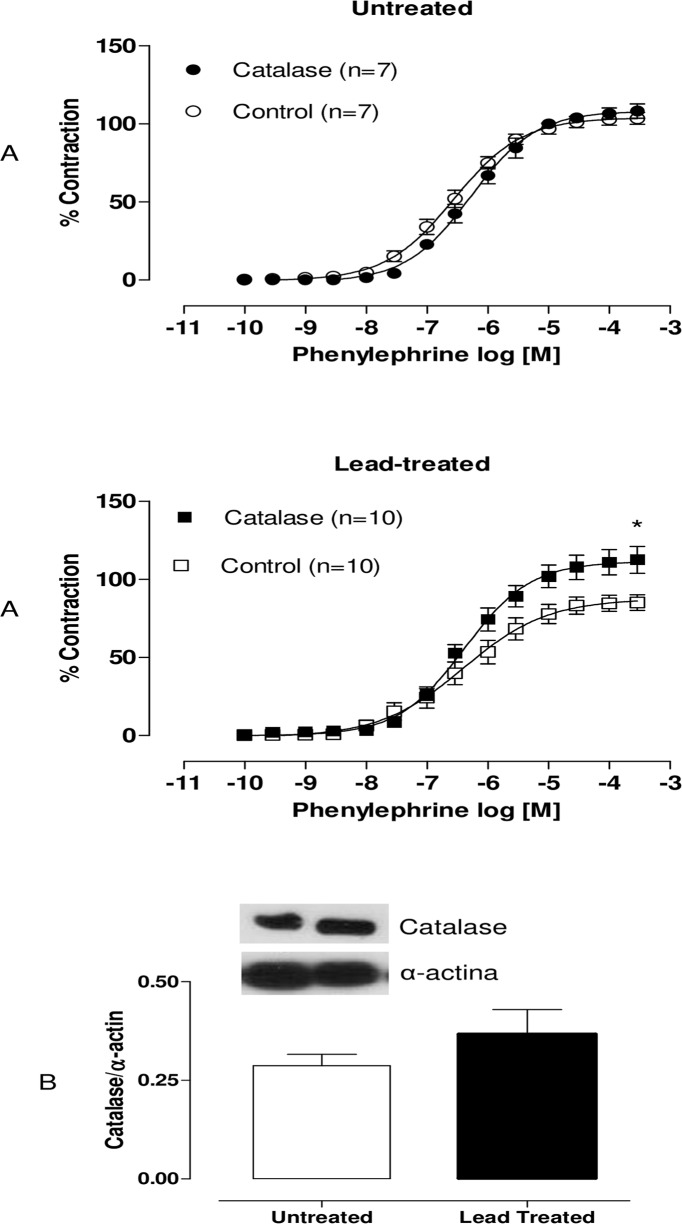
Effects of the catalase in endothelium-intact aortic segments from both untreated and lead-treated rats (A). Effects of the hydrogen peroxide scavenger, catalase (1000 U/ml), on the concentration-response curves of phenylephrine in endothelium-intact aortic segments from both untreated and lead-treated rats. Densitometry of catalase Western blots in aortas from untreated and lead-treated rats (B). Representative blots are also shown. Each point represents the mean ± SEM.*p < 0.05 versus the corresponding control by Student’s t-test. The number of animals used is indicated in parentheses.

Beginning with the hypothesis that hydrogen peroxide acts on aortic rings and induces a vasodilatory response in the lead-treated group, we incubated aortic rings with catalase (1000 U/mL), a hydrogen peroxide scavenger. This drug incubation increased the vasoconstrictor response induced by phenylephrine in the aortas of lead-treated rats, but it not affect the responses of aortas from untreated rats (Fig. 4A).

A Western blot analysis of catalase was performed to determine that the increase in hydrogen peroxide was the result of the decreased expression of this enzyme. However, our results showed no differences between the groups (Fig. 4B).

**Fig 5:**
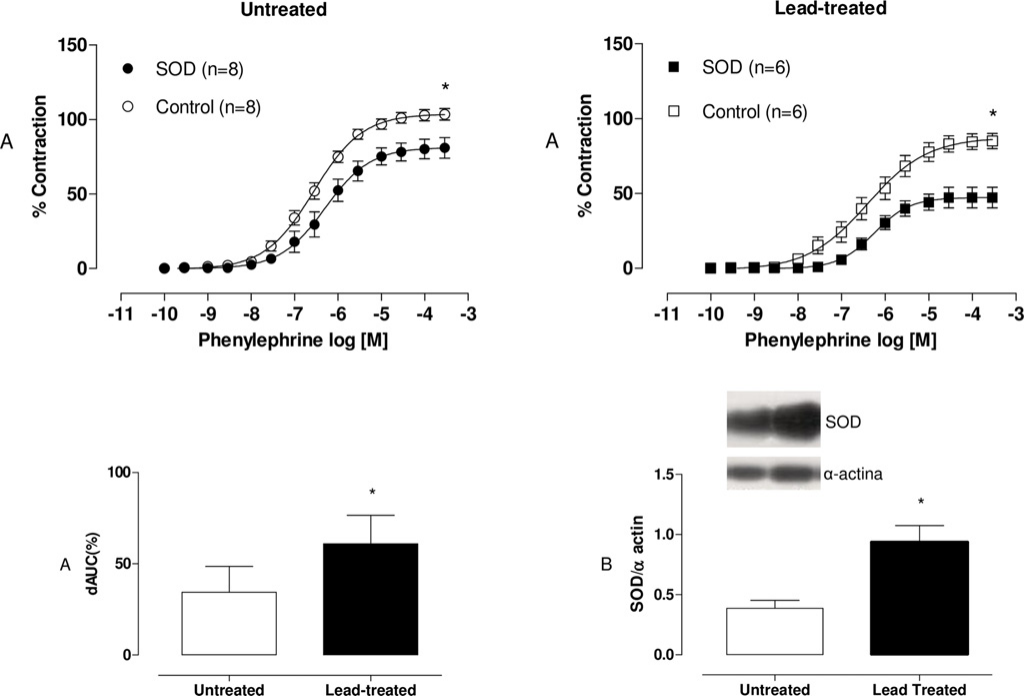
Effects of the SOD in endothelium-intact aortic segments from both untreated and lead-treated rats (A). Effects of the superoxide anion scavenger, SOD (150 U/ml), on the concentration-response curves of phenylephrine in endothelium-intact aortic segments from both untreated and lead-treated rats. The graph demonstrates differences in the areas under the concentration-response curves (dAUC) between the groups. Densitometry of SOD Western blots in aortas from untreated and lead-treated rats (B). Representative blots are also shown. Each point represents the mean ± SEM. *p <0.05 versus the corresponding control by Student’s t-test. The number of animals used is indicated in parentheses.

We performed incubation with superoxide dismutase (150 U/mL). SOD caused reductions in the maximum response to phenylephrine in the arteries of both groups. However, values of dAUC demonstrated that these effects were greater in preparations from lead-treated rats than in preparations from untreated rats (Fig. 5A). This result suggests the following two hypotheses: either SOD expression is increased in animals treated with lead, or the levels of superoxide anions involved in the contractile response to phenylephrine are greater in preparations from lead-treated rats than in preparations from untreated rats.

**Fig 6:**
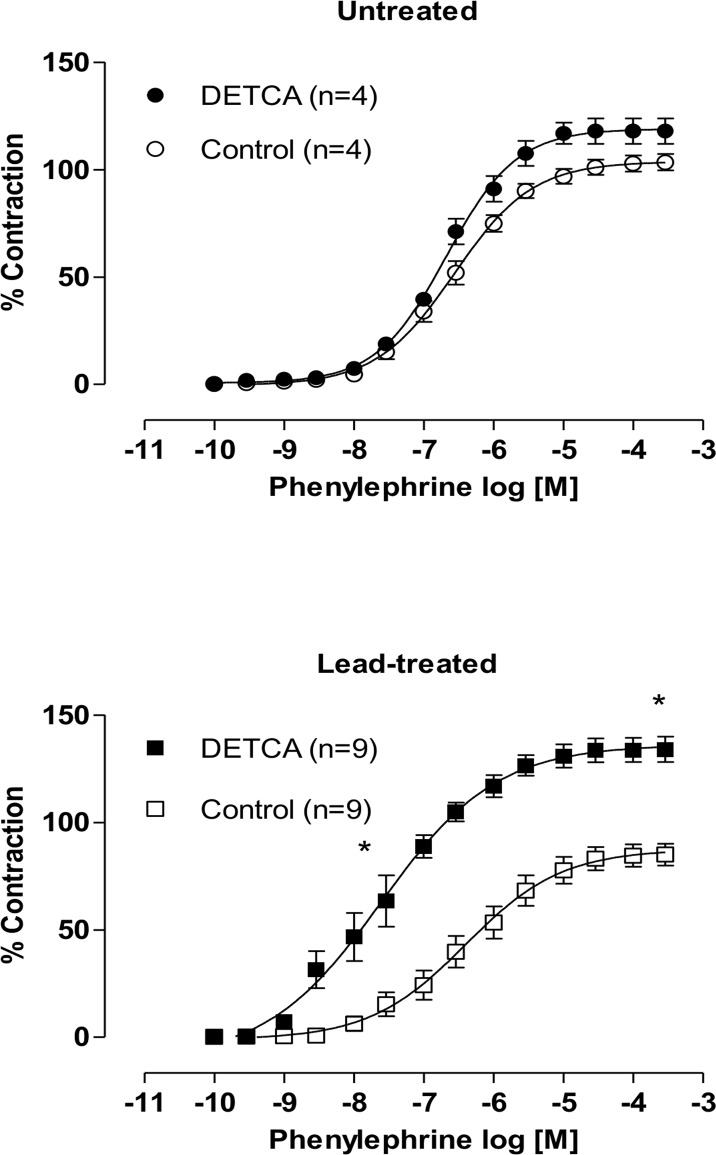
Effects of the DETCA in endothelium-intact aortic segments from both untreated and lead-treated rats. Effects of the inhibitor Cu/Zn superoxide dismutase, DETCA (0.5 mM), on the concentration-response curves of phenylephrine in endothelium-intact aortic segments from both untreated and lead-treated rats. Each point represents the mean ± SEM. *p < 0.05 versus the corresponding control by Student’s t-test. The number of animals used is indicated in parentheses.

In order to investigate whether SOD expression was affected in lead-treated rats, we performed Western blot analyses. They demonstrated increased protein expression of SOD Cu/Zn (Fig. 5B). We performed incubation with DETCA (0.5 mM), an inhibitor of Cu/Zn superoxide dismutase that increased both vasoconstrictor responses and phenylephrine sensitivity in the aortic rings of animals treated with lead (Fig. 6). These results suggest that superoxide anion levels and endogenous Cu/Zn SOD activity are both increased in lead-treated animals, which may contribute to increased hydrogen peroxide levels.

**Fig 7:**
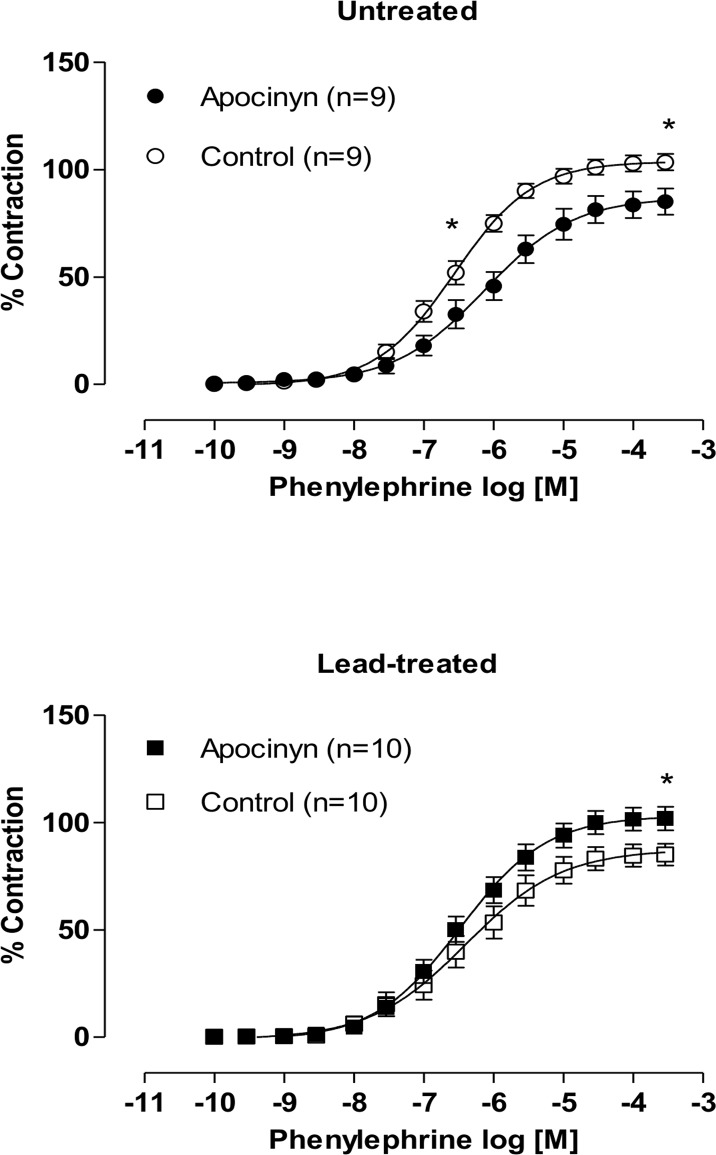
Effects of the apocynin in endothelium-intact aortic segments from both untreated and lead-treated rats. Effects of the specific NAD(P)H oxidase inhibitor, apocynin (0.3 mM), on the concentration-response curves of phenylephrine in endothelium-intact aortic segments from both untreated and lead-treated rats. Each point represents the mean ± SEM. *p < 0.05 versus the corresponding control by Student’s t-test. The number of animals used is indicated in parentheses.

In order to investigate whether NADPH oxidase was associated with increased levels of superoxide anions in lead-treated animals, we used apocynin (0.3 mM), an NADPH oxidase inhibitor. Apocynin reduced phenylephrine’s responsiveness in the untreated group, whereas the opposite response was observed in lead-treated animals, suggesting that this pathway may be involved in the production of superoxide anions and the subsequent production of hydrogen peroxide (Fig. 7).

### Participation of potassium channels in the vasodilator response to phenylephrine

**Fig 8:**
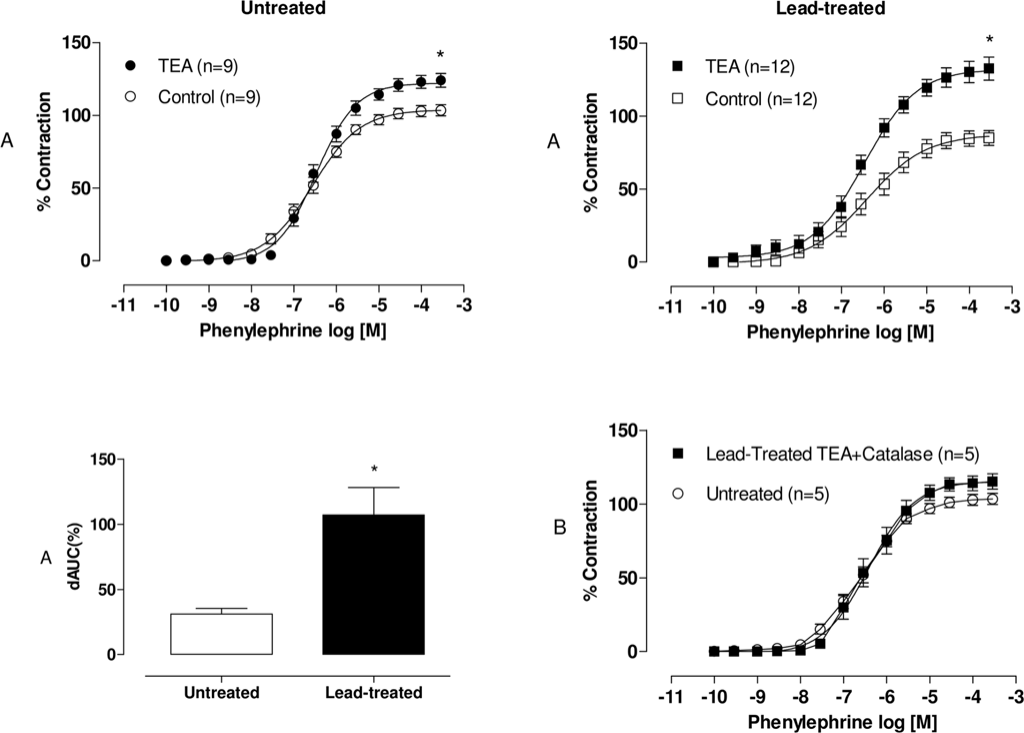
Effects of the TEA in aortic segments from both untreated and lead-treated rats (A). Effects of the nonselective K^+^ channel blocker, TEA (2 mM) on the concentration-response curves of phenylephrine in endothelium-intact aortic segments from both untreated and lead-treated rats. The inset shows differences in dAUC in the presence and absence of TEA. Effects of the co-incubation of catalase/TEA in aortic segments from untreated and lead-treated rats (B). Effects of the co-incubation of catalase (1000 U/mL) with TEA (2 mM) on the concentration-response curves of phenylephrine in endothelium-intact aortic segments from both untreated and lead-treated rats. Each point represents the mean ± SEM. *p < 0.05 versus the corresponding control by Student’s t-test. The number of animals used is indicated in parentheses.

Hydrogen peroxide-induced relaxation involves multiple pathways, including the hyperpolarization of vascular smooth muscle cells via potassium channel activation [16]. In order to investigate the involvement of potassium channels in the vasodilator response to phenylephrine, we used TEA (2 mM), a nonselective K^+^ channel blocker. TEA potentiated the vasoconstrictor response induced by phenylephrine in aortic segments from both groups, but these effects were greater in preparations from lead-treated rats than in preparations from untreated rats, as illustrated by the dAUC values (Fig. 8A).

To confirm our hypothesis that hydrogen peroxide acts on potassium channels and induces a vasodilatory response in animals treated with lead, we incubated catalase (1000 U/mL) with TEA (2 mM). The co-incubation abolished the vasodilatory effect observed in the lead-treated group (Fig. 8B).

## Discussion

The major findings of this study indicate that chronic treatment with low concentrations of lead increases systolic arterial blood pressure and decreases the contractile responses induced by phenylephrine in rat aortas. This response is endothelium-dependent and results from increased hydrogen peroxide concentrations and action on potassium channels. This finding may be explained by the compensatory up-regulation of superoxide dismutase (SOD), which catalyzes dismutation of superoxide anions and increases hydrogen peroxide levels.

The blood concentration of lead in the treated group was approximately 8 μg/dL, whereas the control group’s values were below the apparatus’ limits of detection. The Agency for Toxic Substances and Disease Registry (ATSDR) lists a reference blood concentration of lead of 60 μg/dL [2,17]. The Centers for Disease Control and Prevention consider blood lead concentrations greater than or equal to 30 μg/dL to be elevated in adults, whereas blood lead concentrations greater than or equal to 10 μg/dL are considered elevated in infants and children [18]. However, our study demonstrates that chronic exposure to low concentrations of lead, which results in blood lead levels lower than those considered safe, is harmful and causes cardiovascular alterations.

In spite of the reduction in aortic vascular reactivity, we observed increases in blood pressures on the 7^th^ day of treatment that persisted until the conclusion of treatment. This result may be related to an increase in sympathetic activity in vivo. Previous reports have indicated that lead exposure induces sympathetic hyperactivity by acting on central and peripheral sympathetic junctions, which increases the responsiveness of alpha 2-adrenoreceptors, cardiac and vascular beta-adrenergic receptors and dopaminergic receptors to stimulation. Furthermore, lead treatment increases plasma levels of noradrenaline and adrenaline [19,20].

We observed a reduction in aortic ring reactivity to phenylephrine following thirty days of lead exposure. The reduction in vascular reactivity to phenylephrine was accompanied by a concomitant increase in the endothelial modulation of such responses. We observed that L- NAME increased the reactivity of phenylephrine in both experimental groups; however, dAUC values demonstrated that the role played by nitric oxide in the contractile response to phenylephrine did not differ between the groups. Several reports have also suggested that exposure to lead increases NO production [8,21], which contributed to reductions in reactivity to phenylephrine in aortic rings. This result was associated with increased iNOS protein expression and increased eNOS phosphorylation at Ser1177, the primary regulator of NO production [22,23,8]. The increased NO may open K^+^ channels and contribute to the increased negative modulation of phenylephrine-induced contraction [16]. NO also indirectly activates BK_Ca_ channels by preventing the formation of an endogenous inhibitor of these channel [24,25].

In addition to NO, other endothelium-derived factors such as hydrogen peroxide hyperpolarize and relax underlying smooth muscle cells by activating potassium channels, which may be accomplished directly or following soluble guanylyl cyclase stimulation [16]. Hydrogen peroxide is produced in endothelial and smooth muscle cells from the superoxide anion, primarily by superoxide dismutase [26], and is converted into water and molecular oxygen by catalase [27]. Depending on the blood vessel, the presence of endothelium, the experimental conditions or the concentrations studied, hydrogen peroxide exerts either dilator or constrictor effects [16]. Incubation with catalase increased the vasoconstrictor response induced by phenylephrine in the aortas of lead-treated rats but did not increase the response in the aortas of untreated rats, which suggests increases in the concentrations of hydrogen peroxide in animals treated with lead. Changes in the protein expression of catalase may explain the increases in hydrogen peroxide found in this study. However, no differences between the lead-treated and untreated groups were found.

Several reports have demonstrated that lead-mediated cardiovascular toxicity is strongly associated with oxidative stress-induced [5,28,29] ROS production, indicating that oxidative stress plays an important role in endothelial dysfunction, which is generally associated with cardiovascular disease [27]. Oxidative stress may occur as a result of increased ROS generation or depressed antioxidant pathways or both [30]. We observed significant decreases in the vascular reactivity of the aortic rings of lead-treated animals in the presence of SOD, which is indicative of either increased production of superoxide anions or greater activity of this enzyme. Our hypothesis was confirmed by increases in Cu/Zn-SOD protein expression and increased activity of SOD, which was detected via incubation with DETCA.

However, both decrease [2] and increase of antioxidant reserves [12] mediated by free radicals has been reported. Farmand and collaborators (2005) demonstrated that lead-treated animals exhibited up-regulations in the activity of several antioxidant enzymes as a compensatory response to lead exposure. However, other enzymes did not compensate in the face of oxidative stress, suggesting an antioxidant/oxidant imbalance [12].

Lead is described as an inducer of free radical production [2]. Reactive oxygen species contribute to disease conditions such as inflammation, ischemia-reperfusion injury, atherosclerosis, diabetes mellitus, and hypertension [31,32]. Several epidemiological studies involving workers with high lead exposure have reported a relationship between lead exposure and markers of oxidative stress [33,34].

Recent studies from our laboratory observed greater local superoxide anion production in aortic rings from lead-treated rats and an increased contractility [5]. Our findings, however, show that the blockade of the anion’s production by apocynin causes a reduction in reactivity based on phenylephrine dose-response curves. Although discrepant from our previous findings the model used by Silveira et al., (2014) is based on intramuscular injection of lead, where in the first day of treatment was applied a "attack dose" followed by "maintenance doses". In our study, lead administered orally in drinking water, also promoted blood cumulative concentrations. Thus, these two models of treatment, were able to promote different blood concentrations of lead, which might explain the difference in results.

These findings suggest that NADPH oxidase is also a putative site of action for lead to increase free radical generation [35]. In this study, we observed increases in vasoconstrictor responses in rings incubated with apocynin in the lead-treated group, suggesting that NADPH oxidase may be involved in the production of superoxide anions and the subsequent production of hydrogen peroxide.

Other approaches facilitated transient increases in superoxide production, followed by rises in hydrogen peroxide production in cultured human coronary endothelial and vascular smooth muscle cells following 60 h of lead acetate (1 ppm) treatment. This response was accompanied by a compensatory increase in Cu/Zn-SOD protein expression in the same cells. The short-term exposure to lead resulted in significant elevations of both superoxide and hydrogen peroxide, whereas chronic lead exposure resulted in the elevation of hydrogen peroxide anions but not superoxide anions [13].

Potassium channels in arterial smooth muscle cells are important modulators of blood vessel diameter. The opening of K^+^ channels in the cell membrane of arterial smooth muscle cells increases K^+^ efflux, which causes membrane potential hyperpolarization. This closes voltage-dependent Ca2^+^ channels, which decreases Ca2^+^ entry and leads to vasodilation [36]. We demonstrated that TEA, a K^+^ channel blocker, potentiated the response to phenylephrine in aortic segments from both groups, but these effects were greater in preparations from lead-treated rats than in preparations from untreated rats. Furthermore, TEA-catalase co-incubation abolished the vasodilatory effect in the aortas of lead-treated animals, which suggests that hydrogen peroxide acts on potassium channels.

In both human coronary arterioles and cultured human endothelial cells, hydrogen peroxide serves as a key endothelium-derived hyperpolarizing factor and mediates flow-induced dilation through a novel mechanism involving protein dimerization and the activation of protein kinase G (PKG)-Iα, as well as the subsequent opening of smooth muscle BK_Ca_ channels [37,38]. However, questions remain unanswered regarding whether hydrogen peroxide is a diffusible factor that activates potassium channels on smooth muscle cells or an intracellular messenger involved in the activation of endothelial potassium channels [16].

In conclusion, the present study was the first to demonstrate that blood lead concentrations of 8 μg/dL, which are below values established by international legislation, increased blood pressure and decreased phenylephrine-induced vascular reactivity. This effect was associated with oxidative stress, specifically increases in hydrogen peroxide and its actions on potassium channels. These results also demonstrated that low concentrations of lead are an important risk factor for cardiovascular disease.
